# Artificial Intelligence–Aided Diagnosis System for the Detection and Classification of Private-Part Skin Diseases: Decision Analytical Modeling Study

**DOI:** 10.2196/52914

**Published:** 2024-12-27

**Authors:** Wei Wang, Xiang Chen, Licong Xu, Kai Huang, Shuang Zhao, Yong Wang

**Affiliations:** 1 School of Automation Central South University Changsha China; 2 Department of Dermatology Xiangya Hospital Central South University Changsha China; 3 Jinhua Fifth Hospital Jinhua China

**Keywords:** artificial intelligence-aided diagnosis, private parts, skin disease, knowledge graph, dermatology, classification, artificial intelligence, AI, diagnosis

## Abstract

**Background:**

Private-part skin diseases (PPSDs) can cause a patient’s stigma, which may hinder the early diagnosis of these diseases. Artificial intelligence (AI) is an effective tool to improve the early diagnosis of PPSDs, especially in preventing the deterioration of skin tumors in private parts such as Paget disease. However, to our knowledge, there is currently no research on using AI to identify PPSDs due to the complex backgrounds of the lesion areas and the challenges in data collection.

**Objective:**

This study aimed to develop and evaluate an AI-aided diagnosis system for the detection and classification of PPSDs: aiding patients in self-screening and supporting dermatologists’ diagnostic enhancement.

**Methods:**

In this decision analytical modeling study, a 2-stage AI-aided diagnosis system was developed to classify PPSDs. In the first stage, a multitask detection network was trained to automatically detect and classify skin lesions (type, color, and shape). In the second stage, we proposed a knowledge graph based on dermatology expertise and constructed a decision network to classify seven PPSDs (condyloma acuminatum, Paget disease, eczema, pearly penile papules, genital herpes, syphilis, and Bowen disease). A reader study with 13 dermatologists of different experience levels was conducted. Dermatologists were asked to classify the testing cohort under reading room conditions, first without and then with system support. This AI-aided diagnostic study used the data of 635 patients from two institutes between July 2019 and April 2022. The data of Institute 1 contained 2701 skin lesion samples from 520 patients, which were used for the training of the multitask detection network in the first stage. In addition, the data of Institute 2 consisted of 115 clinical images and the corresponding medical records, which were used for the test of the whole 2-stage AI-aided diagnosis system.

**Results:**

On the test data of Institute 2, the proposed system achieved the average precision, recall, and *F*_1_-score of 0.81, 0.86, and 0.83, respectively, better than existing advanced algorithms. For the reader performance test, our system improved the average *F*_1_-score of the junior, intermediate, and senior dermatologists by 16%, 7%, and 4%, respectively.

**Conclusions:**

In this study, we constructed the first skin-lesion–based dataset and developed the first AI-aided diagnosis system for PPSDs. This system provides the final diagnosis result by simulating the diagnostic process of dermatologists. Compared with existing advanced algorithms, this system is more accurate in identifying PPSDs. Overall, our system can not only help patients achieve self-screening and alleviate their stigma but also assist dermatologists in diagnosing PPSDs.

## Introduction

Skin diseases affect 1.9 billion people worldwide and place a burden on patients’ mental health and quality of life [1-3]. Private-part skin diseases (PPSDs) are a group of high-incidence skin diseases that occur in the private parts of the human body (such as breasts, genitals, or anus), including syphilis, genital herpes, Paget disease, and condyloma acuminatum [4-7]. In clinical practice, a patient needs to expose the affected area sufficiently to dermatologists for visual inspection. Under this condition, patients with PPSDs may become nervous and embarrassed due to stigma and may be reluctant to see a doctor, which hinders the early diagnosis of PPSDs [8-11]. Patients may also be unaware of PPSDs, which is another important reason for the delay of the PPSD diagnosis. For example, sometimes patients easily mistake Paget disease for eczema, which may lead to cancer metastasis. Moreover, patients with PPSDs may hide some medical records because PPSDs may be related to sexual infidelity. Therefore, in order to promote the early diagnosis of PPSDs, it is necessary to develop a novel and private artificial intelligence (AI)–aided diagnosis technology.

Among many advanced AI algorithms, convolutional neural networks (CNNs) have developed rapidly and have shown remarkable performance on many computer vision–related tasks [12-16]. CNNs can learn meaningful and robust features directly from data and have been widely used in AI-aided diagnosis of skin diseases [17-22]. For example, Esteva et al [23] trained an end-to-end CNN with a dataset of 129,450 clinical images. The performance of this CNN is comparable to that of dermatologists in two binary classification tasks (keratinocyte carcinoma vs benign seborrheic keratosis and melanoma vs benign nevus) [23]. It is a seminal work in applying AI to skin disease diagnosis. Fink et al [24] used a pretrained GoogleNet Inception_v4 architecture to classify combined naevi and melanomas and achieved better performance than 11 trained dermatologists [24].

Although CNN-based algorithms have shown promising results in the field of skin disease diagnosis, most of them focus on the classification of skin tumors, and none has been developed for the diagnosis of PPSDs. It may be a feasible solution to introduce existing CNN-based algorithms in skin diseases to assist diagnosis of PPSDs. However, directly applying them to PPSDs has the following two issues. First, the skin representation in clinical images of private parts is complex. For example, a large area in clinical images of private parts is occupied by irregular tissues or other visual obstacles (eg, hair and skinfolds) that are irrelevant to disease recognition, which has a negative effect on the recognition performance. Second, the data on PPSDs is scarce because it is difficult to collect the data, which limits the application of CNN-based algorithms in PPSD diagnosis. Because of these two issues, the classification of the clinical images of PPSDs is usually more difficult than that of images in natural scenes [25]. Therefore, it is necessary to develop more effective techniques to improve the classification performance of PPSDs.

In this paper, we make the first attempt at the assistant diagnosis of PPSDs and develop a two-stage AI-aided diagnosis system by simulating the diagnostic process of dermatologists. This system builds a dermatological knowledge graph from skin lesions and medical records to disease diagnosis, aiming to reduce the model’s dependence on data. Therefore, the system can render the diagnosis of PPSDs with only a small amount of data. Unlike other algorithms that directly learn the mapping from original images to diseases, this system recognizes the type, color, and shape of all skin lesions in original images, and combines the recognition results of skin lesions with the medical records to determine diseases. This system simplifies the disease classification problem into the skin lesion classification problem, which eliminates irregular tissues and visual obstacles in original images to a certain degree, thus alleviating the issue of complex skin representation in clinical images.

When our system is applied to dermatological assistant diagnosis, patients with PPSDs can avoid face-to-face diagnosis with dermatologists, thereby relieving their sense of shame and embarrassment. In addition, patients with PPSDs can carry out disease screening in time and fill in the medical records truthfully.

## Methods

### Ethical Considerations

This study is a secondary analysis based on the data generated during the diagnosis and treatment of PPSDs, all of which were approved by the Ethics Committee of Xiangya Hospital (review number 202308636). Before taking photos, all patients signed informed consent forms for imaging data shooting and scientific research analysis. Of course, all of these data were anonymized and the information that exposed the patients’ identities was removed. As it is a necessary step in the diagnosis and treatment of PPSDs, patients who provide data have no additional financial compensation. We ensure that all the images in the written materials published by this study do not contain the identity information of individual participants.

### Dataset Collection and Annotation

For this study, ethics review and institutional review board approval were obtained from 2 participating institutes (Institute 1: Xiangya Hospital of Central South University, and Institute 2: Wuhan No.1 Hospital). This study followed the Helsinki protocol and good clinical practice guidelines [26,27]. For the development and evaluation of our AI-aided diagnosis system, we retrospectively collected clinical images of PPSDs from the two institutes between July 2019 and April 2022. The collection criteria include (1) private parts skin diseases, (2) images do not affect judgment, and (3) informed consent holder. Exclusion criteria include (1) the inability to capture clear images and (2) those who do not agree to disclose images of skin lesions. These processes were reported in accordance with the TRIPOD (Transparent Reporting of a multivariable prediction model for Individual Prognosis or Diagnosis) guidelines. The data collection process can be divided into 2 steps.

The first step was the collection of clinical images and their corresponding medical records for 7 categories of PPSDs: condyloma acuminatum, Paget disease, eczema, pearly penile papules, genital herpes, syphilis, and Bowen disease. Most of the collected cases had pathological confirmation and the remaining cases were unanimously recognized by 3 dermatologists from Institute 1. The medical records included the location of skin lesions, subjective symptoms, medication history, and history of high-risk sexual activities. Through data collection, we have collected 635 clinical images, of which 520 clinical images from Institute 1 were used for system development, and 115 clinical images and their corresponding medical records from Institute 2 were used for system evaluation. Detailed information on the PPSD dataset is provided in Table S1 in Multimedia Appendix 1.

In the second step, we conducted data labeling and cropping on the collected clinical images. To simulate the visual diagnosis process of dermatologists, three dermatologists from Institute 1 were asked to label the clinical images at the bounding box level, where each bounding box represents a skin lesion (see examples later). These labels characterize the appearance of the skin lesions, including type (papule, plaque, nodule, erosion, and vesicle), color (red, brown, and skin color), and shape (rice shape, round, irregular, papillary, and cauliflower). By cropping the bounding boxes from the original images (each may have multiple bounding boxes), we finally obtained 2701 skin lesion images. It is worth noting that among the five types of skin lesions, the amount of data for erosion and vesicle is relatively small, and we added some images of these 2 types of skin lesions from other human body parts.

### AI-Aided Diagnosis System Overview

In the first stage, the multitask detection network consists of a detection model and a multitask classification model. For an input original image, the detection model first uses the feature extraction module together with the region proposal network to extract the region of interests in the image, that is, the skin lesions. Then, we perform the cropping operation. Note that an image may have multiple bounding boxes of skin lesions. Under this condition, the results of the detection model include multiple cropped images of skin lesions. Then, these cropped images are fed into the multitask classification model to further predict the type, color, and shape of skin lesions, together with their confidence scores (probabilities). Afterward, we count the number of skin lesions in each type. The detailed architecture of the multitask detection network can be seen in Section S1 in Multimedia Appendix 1.

In the second stage, to imitate the dermatologists’ diagnostic process (visual diagnosis and inquiry diagnosis), we designed a decision network based on a dermatological knowledge graph (Figure S1 in Multimedia Appendix 1), which was constructed by dermatologists referencing medical guidelines and professional textbooks. The dermatological knowledge graph is composed of 30 decision paths. For each decision path, the decision network encodes the detection results of the first stage with medical records into a vector using predefined encoding rules. Subsequently, the inference result for each decision path is derived by multiplying the elements within the vector. Finally, the probabilities of all decision paths belonging to the same disease are summed and normalized to infer the final decision result. The inference process is shown in Figure S2 and Section S2 in Multimedia Appendix 1.

**Figure 1:**
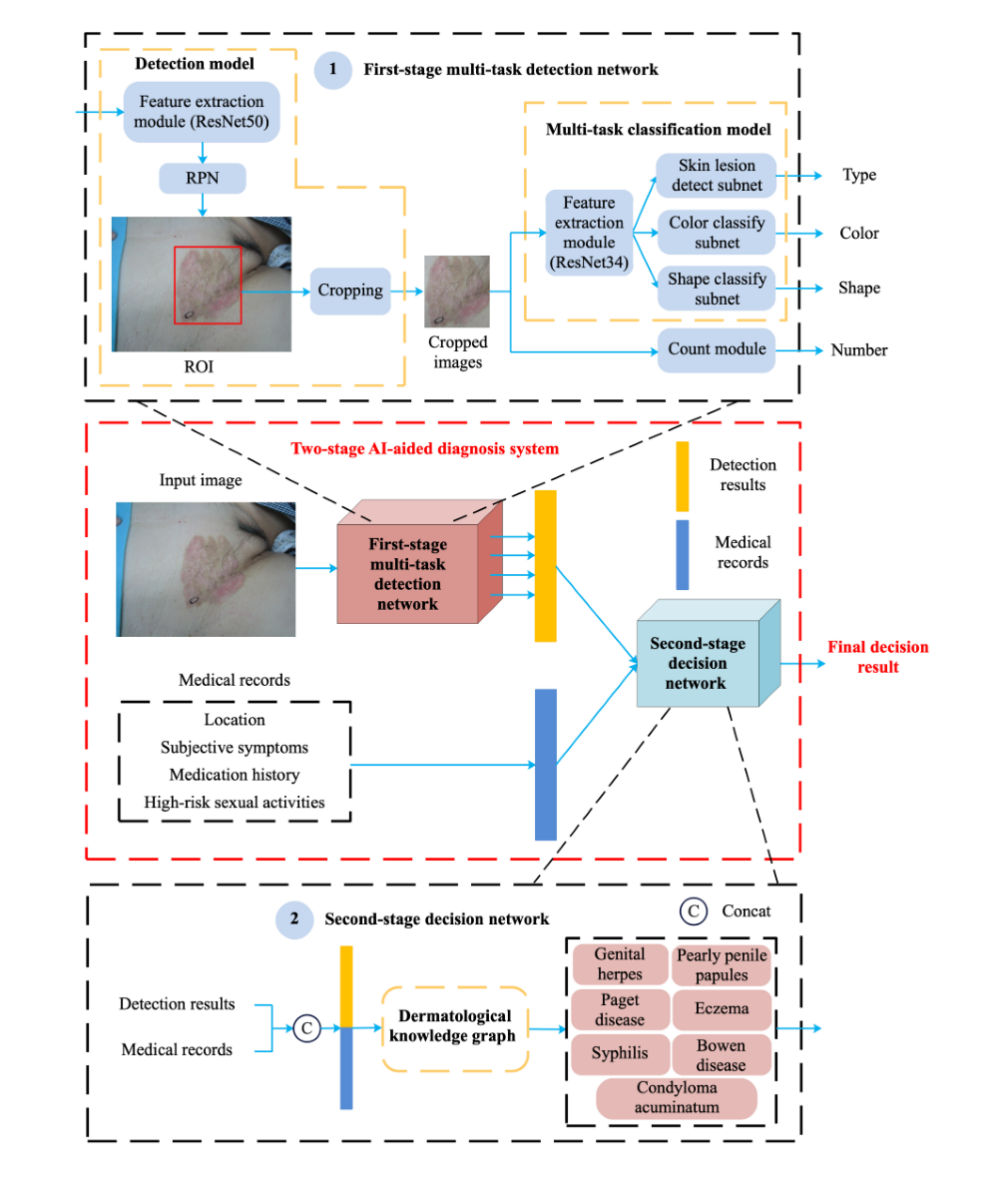
Flowchart of the proposed two-stage AI-aided diagnosis system for PPSDs. AI: artificial intelligence; PPSD: private-part skin disease; ROI: region of interest; RPN: region proposal network.

Inspired by how dermatologists diagnose disease, we proposed a 2-stage AI-aided diagnosis system capable of processing multimodal information, as shown in Figure 1. In the first stage, we trained a multitask detection network to jointly learn the type, color, and shape of skin lesions. In the second stage, we designed a decision network embedded with dermatology expertise, which combined the detection results of the first stage with medical records to obtain the final decision result. Overall, our two-stage system leverages multitask learning to explore skin lesion representations at multiple granularities and introduce dermatology expertise to alleviate the data dependency of deep learning models. The details of the two stages are presented as follows.

### Reader Study Protocol

In order to test whether our system can assist dermatologists in diagnosing PPSDs, we conducted a reader study. The participants in this study included 3 senior dermatologists, 5 intermediate dermatologists, and 5 junior dermatologists. Senior dermatologists refer to dermatologists who have been in medical practice for more than 10 years. Intermediate dermatologists refer to those who have been licensed for more than 3 years. Junior dermatologists are those who have been in medical practice for less than 3 years. The participants were provided with 115 pairs of clinical images and their corresponding medical records as the test set to evaluate the system. They were asked to give the final disease diagnosis for the test set in 2 scenarios. In scenario one, the participants made diagnoses based on the pairs of clinical images and medical records. In scenario two, the participants were additionally given the skin lesion detection results and disease prediction results (with PPSD confidence score) provided by our system, and they were asked to remake their diagnoses. We recorded the performance of the participants in both scenarios and calculated the performance difference between these two scenarios for each PPSD.

### Performance Evaluation Index

To evaluate the first-stage multitask detection network, we used the confusion matrix, receiver operating characteristic (ROC) curve, and area under the ROC curve (AUROC) to test the performance of skin lesion classification. The confusion matrix is used to observe the performance of the multitask classification model in various categories. Each row of the matrix represents the instances in an actual class while each column represents the instances in a predicted class. The ROC curve is the plot of the true positive rate against the false positive rate, at various threshold settings. AUROC measures the entire 2D area underneath the entire ROC curve, and a larger AUROC value represents better classification performance. In addition, for system evaluation, we calculated precision, recall, and *F*_1_-score to measure the PPSD classification performance. These indices are defined as: precision=TP/(TP+FP), recall=TP/(TP+FN), and *F*_1_-score=2×precision×recall/(precision+recall), where TP, TN, FP, and FN are the numbers of true positives, true negatives, false positives, and false negatives, respectively. During the evaluation process, for a positive sample, if the classifier predicts it as a positive sample, it is marked as true positive; otherwise, it is marked as false negative. For a negative sample, if the classifier predicts it as a negative sample, it is marked as true negative; otherwise, it is marked as false positive.

## Results

### Performance Report of the Multitask Detection Network

**Figure 2:**
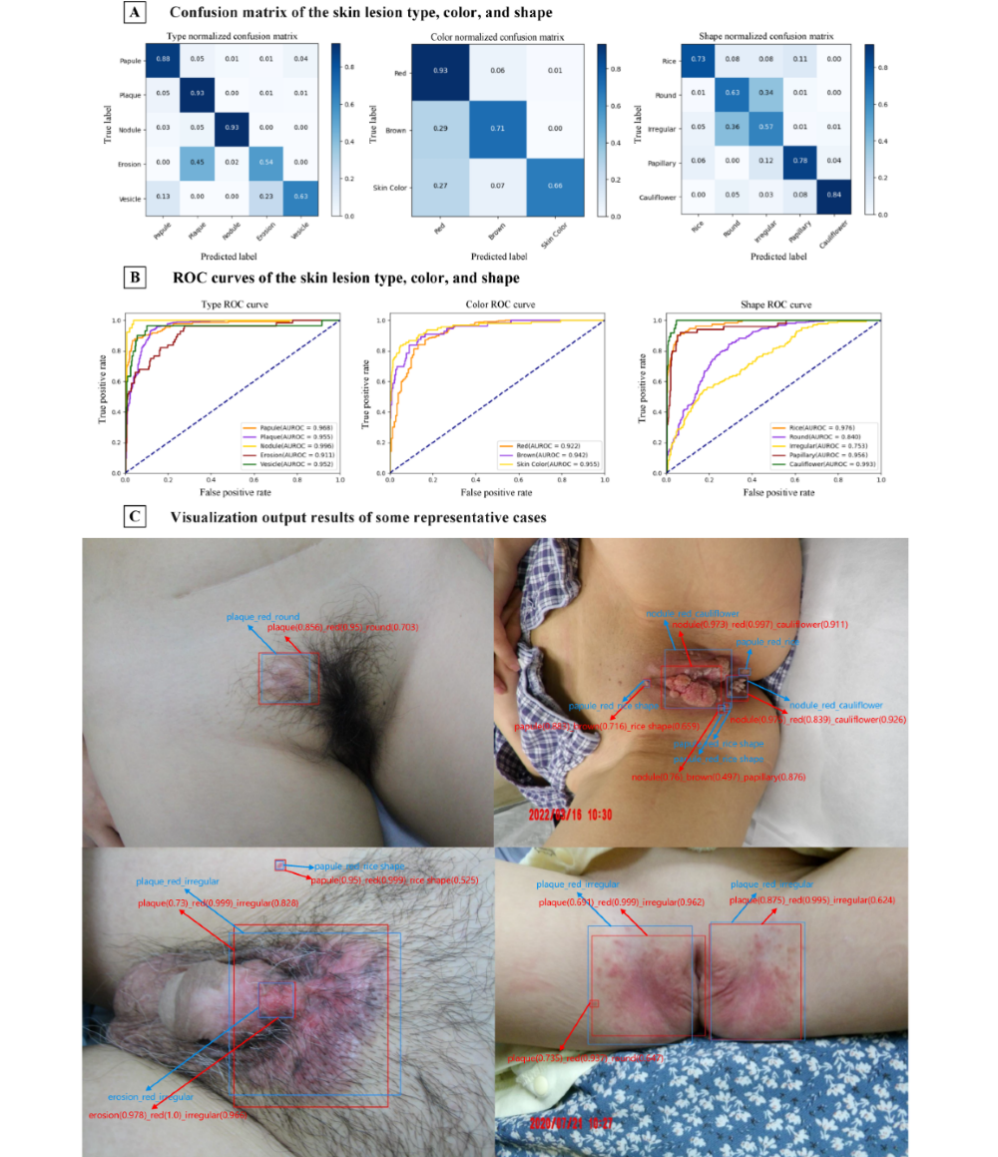
Results of multitask classification model. (A) The confusion matrix. (B) The ROC curve. (C) The visualization results. ROC: receiver operating characteristic.

To verify the performance of the multitask detection network in the first stage, we carried out the classification of the type, color, and shape of skin lesions. From Figure 2A, the results of the confusion matrices show that the network has the highest accuracy on both plaque (0.93) and nodule (0.93) in lesion type recognition. In color and shape recognition, our network achieved the highest accuracy on red (0.93) and cauliflower (0.84), respectively. The results in Figure 2B suggest that the AUROCs of all categories in type and color recognition are higher than 0.9. In particular, the AUROCs of nodule in type recognition and skin color in color recognition are 0.996 and 0.955, respectively. In shape recognition, our network performed well on cauliflower (0.993), rice shape (0.976), and papillary (0.956), but performed relatively poorly on round (0.840) and irregular (0.753). The reason for this observation is elaborated in the discussion.

Figure 2C visualizes the output results of the first-stage multitask detection network in some representative cases. Here, the blue bounding boxes and texts represent the ground truths of the locations of the skin lesions and the ground truths of the labels of the skin lesion type, color, and shape, respectively. The red bounding boxes and texts represent the detection results of this network, and the numbers in brackets denote the confidence scores of the predicted categories.

### Comparison of Existing Algorithms and Proposed System

To demonstrate the effectiveness of the system, we compared it with seven existing advanced deep learning algorithms. Among the 7 algorithms, ResNet101, SENet, SKNet, and Convnext are CNN-based networks, and Deit, Levit, and Swin-twins are transformer-based networks. The results in Table 1 show that the average precision, recall, and *F*_1_-score of our system on seven PPSDs are 0.81, 0.86, and 0.83, respectively, better than those of all the competitors. The system performance during training and validation is shown in Table S2 in Multimedia Appendix 1. In addition, our system achieved the best *F*_1_-score among all the compared algorithms on condyloma acuminatum (0.90), genital herpes (0.94), Bowen disease (0.63), eczema (0.82), Paget disease (0.82), and syphilis (0.78). The comparison of the seven existing methods and the proposed system in computation time is given in Table S3 and Section S3 in Multimedia Appendix 1.

**Table 1:** Precision, recall, and *F*_1_-score of our system and some advanced algorithms for each PPSD^a^.

PPSDs		ResNet101	SKNet	Convnext	Deit	SENet	Levit	Swin-twins	Ours
**Average**									
	F1^b^	0.52	0.59	0.57	0.59	0.59	0.60	0.69	*0.83* ^c^
	R^d^	0.57	0.62	0.59	0.60	0.63	0.61	0.69	*0.86*
	P^e^	0.55	0.62	0.72	0.62	0.64	0.64	0.72	*0.81*
**SP^f^**									
	F1	0.21	0.51	0.22	0.57	0.39	0.40	0.44	*0.78*
	R	0.13	0.38	0.13	0.50	0.25	0.29	0.33	*0.73*
	P	0.60	0.82	*1.00*	0.67	0.86	0.64	0.67	*0.84*
**PD^g^**									
	F1	0.64	0.64	0.77	0.59	0.66	0.71	0.71	*0.82*
	R	0.74	*0.91*	0.87	0.65	0.87	0.87	0.74	0.78
	P	0.57	0.49	0.69	0.54	0.53	0.61	0.68	*0.86*
**EZ** ^h^									
	F1	0.32	0.17	0.36	0.48	0.36	0.34	0.67	*0.82*
	R	0.35	0.12	0.35	0.41	0.35	0.35	0.82	*0.94*
	P	0.29	0.29	0.38	0.58	0.38	0.33	0.56	*0.73*
**BD^i^**									
	F1	0.10	0.31	0.45	0.13	0.19	0.33	0.38	*0.63*
	R	0.06	0.25	0.44	0.13	0.13	0.25	0.38	*0.56*
	P	0.20	0.40	0.47	0.14	0.40	0.50	0.38	*0.71*
**PPP^j^**									
	F1	0.91	*1* *.00*	*1* *.00*	0.91	*1.00*	*1* *.00*	0.89	0.91
	R	1.00	1.00	1.00	1.00	1.00	1.00	0.80	*1* *.00*
	P	0.83	*1.00*	*1* *.00*	0.83	*1* *.00*	*1* *.00*	*1* *.00*	0.83
**GH^k^**									
	F1	0.80	0.78	0.55	0.77	0.78	0.71	0.93	*0.94*
	R	0.75	0.88	0.38	0.63	0.88	0.63	0.88	*1* *.00*
	P	0.86	0.70	*1* *.00*	*1* *.00*	0.70	0.83	*1.00*	0.89
**CA^l^**									
	F1	0.67	0.71	0.64	0.70	0.75	0.71	0.79	*0.90*
	R	0.95	0.82	0.95	0.86	0.95	0.91	0.86	*1.00*
	P	0.51	0.62	0.48	0.59	0.62	0.59	0.73	*0.81*

^a^PPSD: private-part skin disease.

^b^F1: *F*_1_-score.

^c^Values in italics format indicate optimal values under the corresponding indicators.

^d^R: recall.

^e^P: precision.

^f^SP: syphilis.

^g^PD: Paget disease.

^h^EZ: eczema.

^i^BD: Bowen disease.

^j^PPP: pearly penile papules.

^k^GH: genital herpes.

^l^CA: condyloma acuminatum.

### Reader Study Performance

In this study, the diagnostic performance (including precision, recall, and *F*_1_-score) of the 3 groups of dermatologists was evaluated in 2 scenarios (first without and then with system support). From Table 2, in terms of the average *F*_1_-score, our system (0.83) outperformed the junior (0.57) and the intermediate dermatologists (0.78) in scenario 1, and was comparable to the senior dermatologists (0.85). Furthermore, with the support of the system (scenario 2), the dermatologists improved their diagnostic performance. Among the 3 groups of dermatologists, the junior group showed the greatest improvement, and their average precision, recall, and *F*_1_-score increased by 17%, 14%, and 16%, respectively. In particular, the *F*_1_-score of genital herpes had the largest increase of 41%. For the intermediate group, the average precision, recall, and *F*_1_-score all increased by about 7%. The senior group showed the smallest improvement in diagnostic performance, with the average precision, recall, and *F*_1_-score increasing by 2%, 5%, and 4%, respectively.

**Table 2:** Classification performance of the three levels of dermatologists in the first and second scenarios^a^.

Disease			Junior	Junior+AI^b^	Junior-∆^c^	Intermediate	Intermediate+AI	Intermediate-∆	Senior	Senior+AI	Senior-∆	Ours
**Average**												
	F1^d^	0.57		0.73	0.16	0.78	0.85	0.07	0.85	0.89	0.04	0.83
	R^e^	0.61		0.75	0.14	0.78	0.85	0.07	0.85	0.90	0.05	0.86
	P^f^	0.58		0.75	0.17	0.80	0.88	0.08	0.88	0.90	0.02	0.81
**SP^g^**												
	F1	0.67		0.75	0.08	0.78	0.81	0.03	0.87	0.88	0.01	0.78
	R	0.65		0.68	0.03	0.75	0.74	–0.01	0.88	0.85	–0.03	0.73
	P	0.70		0.85	0.15	0.83	0.96	0.13	0.85	0.92	0.07	0.84
**PD^h^**												
	F1	0.46		0.64	0.18	0.75	0.83	0.08	0.86	0.88	0.02	0.82
	R	0.37		0.55	0.18	0.67	0.82	0.15	0.83	0.84	0.01	0.78
	P	0.63		0.78	0.15	0.86	0.85	–0.01	0.91	0.93	0.02	0.86
**EZ^i^**												
	F1	0.68		0.71	0.03	0.68	0.77	0.09	0.79	0.89	0.10	0.82
	R	0.82		0.87	0.05	0.82	0.89	0.07	0.88	0.98	0.10	0.94
	P	0.58		0.61	0.03	0.58	0.70	0.12	0.72	0.81	0.09	0.73
**BD^j^**												
	F1	0.37		0.61	0.24	0.66	0.75	0.09	0.77	0.74	–0.03	0.63
	R	0.34		0.56	0.22	0.62	0.67	0.05	0.70	0.67	–0.03	0.56
	P	0.43		0.67	0.24	0.76	0.85	0.09	0.90	0.86	–0.04	0.71
**PPP^k^**												
	F1	0.64		0.77	0.13	0.95	0.98	0.03	1.00	1.00	0.00	0.91
	R	0.88		0.80	–0.08	1.00	0.96	–0.04	1.00	1.00	0.00	1.00
	P	0.51		0.78	0.27	0.91	1.00	0.09	1.00	1.00	0.00	0.83
**GH^l^**												
	F1	0.36		0.77	0.41	0.73	0.91	0.18	0.75	0.94	0.19	0.94
	R	0.33		0.83	0.50	0.70	0.88	0.18	0.67	0.96	0.29	1.00
	P	0.48		0.76	0.28	0.77	0.96	0.19	0.90	0.93	0.03	0.89
**CA^m^**												
	F1	0.80		0.88	0.08	0.89	0.90	0.01	0.94	0.92	–0.02	0.90
	R	0.89		0.98	0.09	0.93	0.98	0.05	1.00	1.00	0.00	1.00
	P	0.73		0.80	0.07	0.86	0.83	–0.03	0.88	0.85	–0.03	0.81

^a^The dermatologists included 3 senior dermatologists, 5 intermediate dermatologists, and 5 junior dermatologists.

^b^AI: artificial intelligence.

^c^∆ indicates the difference in each evaluation index between two scenarios (dermatologist and dermatologist + AI).

^d^F1: *F*_1_-score.

^e^R: recall.

^f^P: precision.

^g^SP: syphilis.

^h^PD: Paget disease.

^i^EZ: eczema.

^j^BD: Bowen disease.

^k^PPP: pearly penile papules.

^l^GH: genital herpes.

^m^CA: condyloma acuminatum.

## Discussion

### Principal Results

In our study, to help the early diagnosis of PPSDs, we established the first PPSD dataset and developed the first AI-aided diagnosis system of PPSDs. The main finding was that compared with existing advanced classification algorithms, our 2-stage AI-aided diagnosis system achieved more accurate classification performance of PPSDs and detected all the skin lesions in images.

Interpretability is an important factor in AI-aided diagnosis, as it can help clinicians understand the predictions of AI algorithms and enable better human-machine collaboration in clinical practice. In the first stage, our system could detect all the skin lesions in images and give visualization results, which indicates the interpretability of our system to predict skin lesions. In addition, in the second stage, our system simulates the diagnosis process of dermatologists, inferring PPSDs based on the dermatological knowledge graph. Since the dermatological knowledge graph is in line with the clinical routine of dermatologists, our system is interpretable in the prediction of PPSDs.

AI has been proven to be applicable in the diagnosis, treatment, and efficacy prediction of skin diseases. Huang et al [28] used an AI-based approach for assessing the severity of psoriasis, which trains end-to-end with only images and severity scores [28]. They also demonstrated that the severity score predicted by the AI model is close to the Psoriasis Area and Severity Index score diagnosed by experienced dermatologists. Philips et al [29] reported that AI-based classification methods not only outperform human experts in the diagnosis of pigmented skin diseases but also further improve diagnostic accuracy.

During patient consultations, visual inspection is the first step of skin disease diagnosis. The lesion-affected private parts need to be fully exposed to dermatologists, which causes embarrassment for patients and further delays medical treatment, thereby significantly impacting patients’ mental health [30,31]. The system developed in this study avoids the stigma of exposing private parts to strangers, allowing patients to undergo dermatological inspections with more comfort and ease. It should be noted that the purpose of this system is to assist patients in self-screening, not to replace dermatologists. Additionally, in the future, remote medical treatment can be used for digital PPSD treatment, further reducing the degree of medical delays [32,33].

It can be seen from Table 2 that in terms of *F*_1_-score, the system performed poorly for Bowen disease (0.63). The reason is that the dermatological knowledge graph in the second stage considers the shape of the plaque (irregular or round) as the key to the diagnosis of Bowen disease, while the multitask detection network in the first stage has poor recognition performance on irregular and round classifications. It is not surprising that our system performed poorly in recognizing the shape of plaque. In fact, dermatologists also find it challenging to accurately determine the shape of lesions when annotating images of Bowen disease. When assisting dermatologists in diagnosing PPSDs, our system improved the diagnostic performance of all the dermatologists, demonstrating the feasibility of our system in AI-supported image analysis. In particular, with the assistance of our system, the diagnostic performance of dermatologists in the junior group has been improved, basically helping them to reach the ability of dermatologists in the intermediate group. Regarding Paget disease, an extremely serious skin tumor, the *F*_1_-score of dermatologists in the junior group is improved by 18%. The above results preliminarily suggest that our system can help dermatologists in primary hospitals to diagnose PPSDs, and it can also be used as a reference when dermatologists have disagreements.

**Figure 3:**
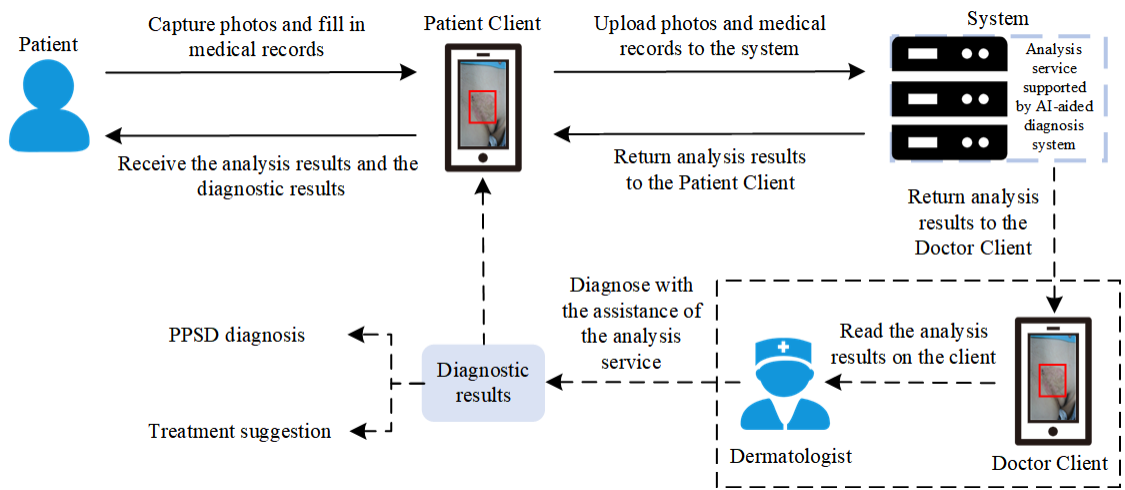
The routine clinical workflow of our two-stage AI-aided diagnosis system. AI: artificial intelligence; PPSD: private-part skin disease.

Our 2-stage AI-aided diagnosis system is helpful to assist patients in self-screening, and the results of the system are sent to dermatologists for further confirmation. The workflow is shown in Figure 3. First, patients with PPSDs can take a photo of the skin lesion, fill in the medical records through the patient client in the mobile phone, and upload them to the system. Then, the system detects all the skin lesions (type, color, and shape) in the photo and combines them with the medical records to generate the preliminary analysis results. Afterward, the preliminary analysis results are sent to dermatologists for further diagnosis. By combining patient information with the analysis results given by the system, dermatologists make the final diagnostic results and treatment suggestions.

### Limitations

This study has some limitations. First, the volume of our data was limited due to the difficulty of data collection on PPSDs. Second, we only included seven categories of PPSDs, so some other diseases were not considered. Third, the decision network in the second stage of our system was designed based on a knowledge graph of dermatology expertise, which consists of a series of directed decision paths. Since a single clinical image cannot fit all decision paths, certain PPSD confidence scores will be 0. As a result, the value of AUROC cannot be properly calculated. Fourth, the variety of datasets was insufficient because we could only collect data from 2 sources. Finally, the diagnostic performance of our system for Bowen disease was not satisfactory due to the difficulty of data labeling. Overall, further efforts should be made to overcome these limitations.

### Conclusions

In this paper, we developed a two-stage AI-aided diagnosis system for PPSDs. Different from existing methods that directly learn image-to-disease mapping, the developed system simulated a dermatologist’s diagnostic process by first identifying skin lesions, and then inferring diseases based on the skin lesion identification results and medical records. The system addressed the issues of complex skin representation in the images of PPSDs and data dependence. Compared with existing advanced algorithms, this system was more clinically relevant and performed better. In addition, the results of the reader study suggest that our system can improve the performance of dermatologists in diagnosing PPSDs. In practical applications, our system has the potential to alleviate the stigma of patients with PPSD and avoid treatment delays.

## Appendix

Multimedia Appendix 1 Additional materials.

## Abbreviations

AI: artificial intelligence
AUROC: area under the receiver operating characteristic curve
CNN: convolutional neural network
PPSD: private-part skin disease
ROC: receiver operating characteristic
TRIPOD: Transparent Reporting of a multivariable prediction model for Individual Prognosis or Diagnosis

## Data Availability

The datasets generated or analyzed during this study are available from the corresponding author upon reasonable request.
