# Supplementary material for: Artificial Intelligence–Aided Diagnosis System for the Detection and Classification of Private-Part Skin Diseases: Decision Analytical Modeling Study
Source: J Med Internet Res. 2024 Dec 27;26:e52914. doi: 10.2196/52914 (PMC11724214; doi:10.2196/52914)
Supplement: Multimedia Appendix 1 [file jmir_v26i1e52914_app1.docx]

**Section S1.** Architecture of the Multi-task Detection Network

**Section S2.** Workflow of the Decision Network

**Section S3.** Computation Time of Different Methods

**Figure S1.** Overview of the Dermatological Knowledge Graph

**Figure S2.** Inference Process of the Dermatological Knowledge Graph

**Figure S3.** Examples of Representative Skin Lesion Images

**Table S1.** Detailed Information of the PPSD Data Set

**Table S2.** Performance during Training and Validation

**Table S3.** Computation Time of Different Methods

This supplemental material has been provided by the authors to give readers additional information about their work.

**Section S1. Architecture of the Multi-task Detection Network**

1-1) Detection Model

The detection model was used to locate and classify the skin lesions in the input clinical images. First, the input clinical images were resized to 1333×800×3, where 1333, 800, and 3 represent the width, height, and number of channels, respectively. Then, the CNN model ResNet50 with Feature Pyramid Network (FPN) was used for feature extraction, which took the resized image as the input and output five different levels of feature maps. Next, we performed a 1×1 convolution on the feature maps in level 2 to level 5. Afterward, the region proposal network (RPN) was utilized for distinguishing foreground objects and background. RPN put many anchors on the input to scan every region in the image and performed classification and bounding box regression on these anchors to obtain region of interests (ROIs). Finally, 2,701 skin lesion images were cropped according to the obtained ROIs.

1-2) Multi-task Classification Model

The multi-task classification model was used to classify the type, color, and shape of the cropped skin lesion images. First, these cropped images were resized to 224×224×3, where 224, 224, and 3 represent the width, height, and number of channels, respectively. Then, the CNN model ResNet34 was used for feature extraction, and output a 512-dimensional feature vector. Next, this feature vector was fed into three convolutional subnets with the same structure to classify the skin lesion information of type (papule, plaque, nodule, erosion, and vesicle), color (red, brown, and skin color), and shape (rice shape, round, irregular, papillary, and cauliflower), respectively. Each convolutional subnet, composed of two fully-connected layers and a Softmax, computed the probabilities of categories of each skin lesion information. In addition, the common augmentation techniques were used, including random horizontal and vertical flipping. Note that, a transfer learning technique (using the pre-trained weights of ResNet34 on ImageNet) was used to speed up the training process and improve the final performance of the multi-task classification model.

**Section S2. Workflow of the Decision Network**

In the second-stage decision network of our system, the dermatological knowledge graph consisted of 30 decision paths (see eFigure 1), and we could infer a diagnosis result of PPSDs (with PPSD confidence scores) through each decision path. Note that the final diagnosis result was the one with the highest PPSD confidence score. For a certain decision path, we first encoded the corresponding category of each medical record (location of skin lesion/subjective symptoms/medication history/history of high-risk sexual activities) of the original input image as 0 (no) or 1 (yes), which formed a four-dimensional binary vector. Then, we encoded the multi-task classification results (type, color, and shape) and the count results of the first-stage detection network into another four-dimensional vector. Specifically, the first three dimensions were encoded by the probability of the corresponding category of each multi-task classification result. As for the fourth dimension, if the count number was greater than 1, the value of this dimension was encoded as 1 and 0 otherwise. Next, we concatenated these two four-dimensional vectors to form an eight-dimensional vector. Then, we multiplied all the elements in the concatenated vector to obtain the PPSD result (with probability) of this decision path. Afterward, we summed the probabilities of all the decision paths belonging to the same disease, and normalized them to obtain the final confidence score of each disease. Finally, the disease with the maximum PPSD confidence score was taken as the final decision result of this decision network.

**Section S3. Computation Time of Different Methods**

As shown in the Table S3, compared with the 7 existing advanced deep learning algorithms, our proposed system exhibits slightly longer computation time. This is attributed to the comprehensive ability of our system, which not only classifies PPSD diseases but also annotates all types, colors, and shapes of skin lesions present in clinical images. Such comprehensive annotation requires longer time than simple classification tasks. However, we consider that this comprehensive approach is necessary. Despite the longer computation time when compared with the 7 advanced deep learning algorithms, our system offers doctors valuable lesion information, aiding the diagnosis of PPSD diseases with higher reliability. Additionally, this work contributes to the interpretability of deep learning models.

**Figure S1. Overview of the Dermatological Knowledge Graph**


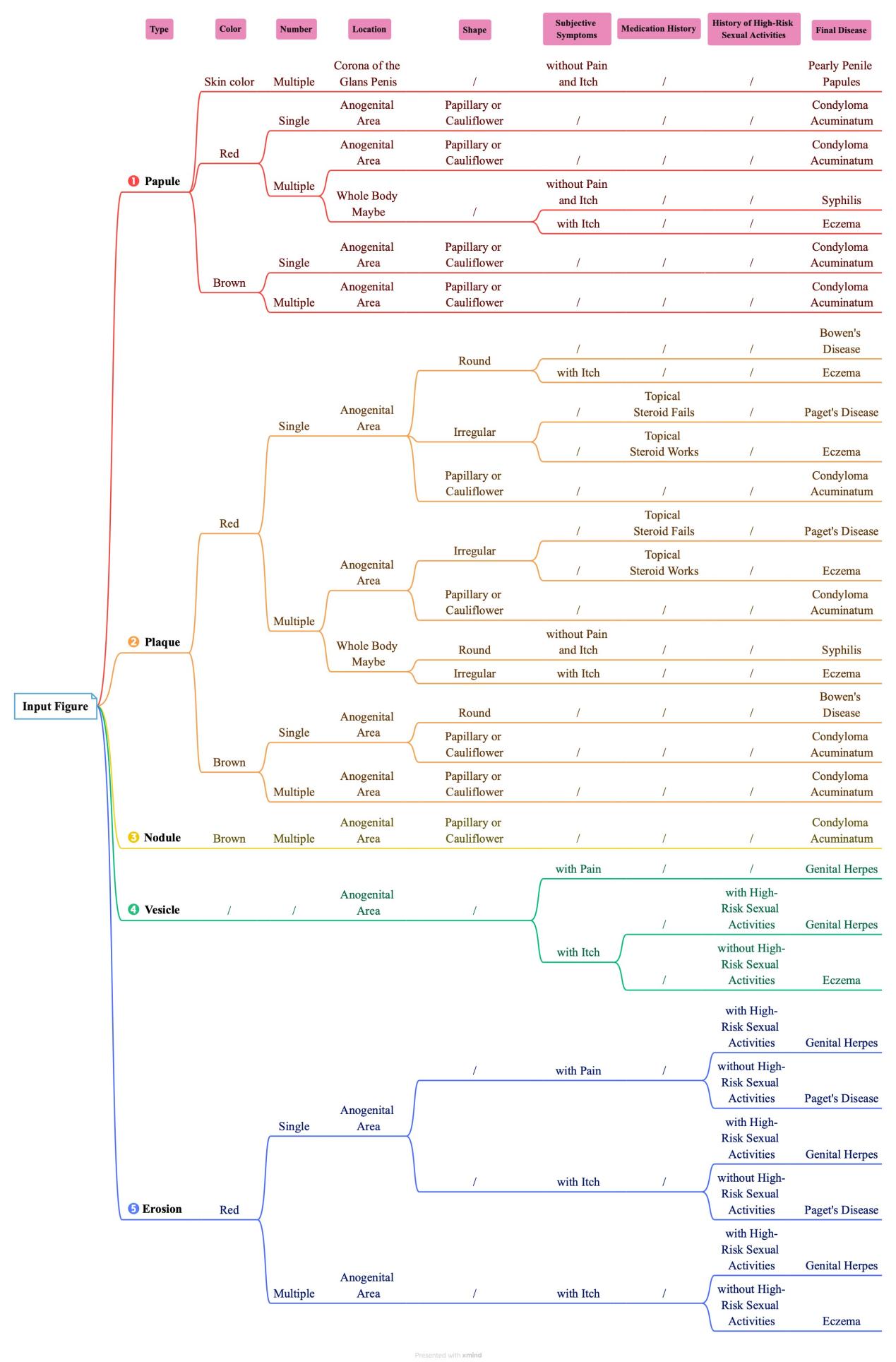


Abbreviations: /, not applicable.

**Figure S2.** **Inference Process of the Dermatological Knowledge Graph**

**Figure S3. Examples of Representative Skin Lesion Images**

**Table S1. Detailed Information of the PPSD Data Set**

|  | **Institute 1** | **Institute 2** |
| --- | --- | --- |
| Diseases |  |  |
| Condyloma Acuminatum | 180 | 22 |
| Paget's Disease | 205 | 23 |
| Eczema | 27 | 17 |
| Pearly Penile Papules | 11 | 5 |
| Genital Herpes | 17 | 8 |
| Syphilis | 11 | 24 |
| Bowen's Disease | 34 | 16 |
| Supplement | 35 | 0 |
| Types of Skin Lesions |  |  |
| Papule | 1012 | 134 |
| Plaque | 968 | 350 |
| Nodule | 305 | 40 |
| Erosion | 264 | 56 |
| Vesicle | 152 | 30 |
| Shapes of Skin Lesions |  |  |
| Rice Shape | 637 | 114 |
| Round | 513 | 268 |
| Irregular | 858 | 141 |
| Papillary Hyperplasia | 505 | 50 |
| Cauliflower-Like | 188 | 37 |
| Colors of Skin Lesions |  |  |
| Red | 1515 | 457 |
| Brown | 766 | 56 |
| Skin Color | 420 | 97 |

**Table S2. Performance during Training and Validation**

|  | Average Precision | Average Recall | Average F1-score |
| --- | --- | --- | --- |
| Training performance | 0.93 | 0.97 | 0.95 |
| Validation performance | 0.81 | 0.86 | 0.83 |

**Table S3. Computation Time of Different Methods**

| Methods | ResNet101 | SKNet | Convnext | Deit | SENet | Levit | Swin-twins | Ours |
| --- | --- | --- | --- | --- | --- | --- | --- | --- |
| Computation time (s) | 28.49 | 29.31 | 26.94 | 26.65 | 29.56 | 30.04 | 30.27 | 78.26 |
